# Widespread ectopic expression of olfactory receptor genes

**DOI:** 10.1186/1471-2164-7-121

**Published:** 2006-05-22

**Authors:** Ester Feldmesser, Tsviya Olender, Miriam Khen, Itai Yanai, Ron Ophir, Doron Lancet

**Affiliations:** 1 Department of Molecular Genetics, Weizmann Institute of Science, Rehovot 76100, Israel; 2 Department of Biological Services, Weizmann Institute of Science, Rehovot 76100, Israel; 3 Present address: Department of Molecular and Cellular Biology, Harvard University, Cambridge, MA 02138, USA

## Abstract

**Background:**

Olfactory receptors (ORs) are the largest gene family in the human genome. Although they are expected to be expressed specifically in olfactory tissues, some ectopic expression has been reported, with special emphasis on sperm and testis. The present study systematically explores the expression patterns of OR genes in a large number of tissues and assesses the potential functional implication of such ectopic expression.

**Results:**

We analyzed the expression of hundreds of human and mouse OR transcripts, via EST and microarray data, in several dozens of human and mouse tissues. Different tissues had specific, relatively small OR gene subsets which had particularly high expression levels. In testis, average expression was not particularly high, and very few highly expressed genes were found, none corresponding to ORs previously implicated in sperm chemotaxis. Higher expression levels were more common for genes with a non-OR genomic neighbor. Importantly, no correlation in expression levels was detected for human-mouse orthologous pairs. Also, no significant difference in expression levels was seen between intact and pseudogenized ORs, except for the pseudogenes of subfamily 7E which has undergone a human-specific expansion.

**Conclusion:**

The OR superfamily as a whole, show widespread, locus-dependent and heterogeneous expression, in agreement with a neutral or near neutral evolutionary model for transcription control. These results cannot reject the possibility that small OR subsets might play functional roles in different tissues, however considerable care should be exerted when offering a functional interpretation for ectopic OR expression based only on transcription information.

## Background

Olfaction, the sense of smell, is mediated by a very large family of olfactory receptors (ORs), whose chemical interaction with a multitude of odorant ligands initiates a cascade of signal transduction events leading to smell perception [1-4]. OR proteins participate also in guiding olfactory sensory neurons axons to their glomerular targets [5], and have been proposed to be involved in the regulation of their own expression [6,7].

The publication of the complete human genome sequence, as well as that of other mammals such as mouse, dog and chimpanzee, allowed the identification of entire OR repertoires in those species via computational data-mining. Currently, 853 human OR genes are known in the human genome [8], 1490 in the mouse genome [9,10], 1493 in the rat genome [11], 971 in the dog genome [12,13] and 1091 ORs in chimpanzee [14]. These are represented in the Human Olfactory Receptor Exploratorium Database (HORDE) and in the Olfactory Receptor DataBase (ORDB) [15]. In addition to this massive information that has been obtained by scrutinizing genome sequence repositories, several publications have provided information on transcription of OR genes in different tissues and species. In mouse, olfactory epithelial ESTs have been sequenced for more than 400 OR genes [16] and a custom mouse OR microarray was used to examine the expression levels of more than 800 genes in olfactory epithelium [17]. In contrast, human OR expression has been investigated for only a small number of genes, as exemplified by the demonstration of transcripts for genes in an OR cluster on human chromosome 17 [18].

ORs are expected to be specifically expressed in the olfactory epithelium, where their expression is also highly regulated by mechanisms which allow each sensory neuron to express a single allele of a single OR gene [19-21]. OR genes are also expressed in the olfactory bulb, where they are specifically localized to the nerve and glomerular layers, potentially related to the targeting of the sensory axons [22,23]. In the present paper we address the question to which degree OR genes are expressed in non-olfactory tissues. We use the term "ectopic" defined as "a biological event or process that occurs in an abnormal location or position within the body" [24]. We note that the term has also been used to describe abnormal expression in malignant tumors, not studied here.

Most of the early reports about ectopic OR expression have been related to testis and germ cells, where several dozens of human and mouse ORs have been shown to be transcribed [25-32]. These results have led to the hypothesis that at least some ORs are involved in mammalian sperm chemotaxis. Evidence for the involvement of human hOR17-4 (OR1D2) [30,33] and mouse MOR267-13 (orthologous to human OR10J5) [34] in sperm chemotaxis has been provided. Another hypothesis proposed that ORs linked to the major histocompatibility complex locus and expressed in testis are implicated in olfaction-driven mate choice [35].

Some human ORs were also shown to be expressed in tongue [36,37], erythroid cells [38] and prostate [39,40]. Some murine ORs have been shown to be expressed ectopically in placenta, brain, peripheral nervous system, colon and fetal liver [41-45]. Because of their broad pattern of tissue expression during development and in adult life, ORs have been proposed to play a role in cell-cell recognition [46]. Despite such reports regarding the potential functions of ectopically expressed ORs, this phenomenon is far from being fully understood and requires further systematic investigation.

Here we report the first systematic global analysis of spatial OR expression patterns in human and mouse. We aim to obtain transcriptional evidence for numerous ORs in a large number of tissues, so as to shed further light on the possible factors influencing OR ectopic expression. To this end, we have collected mouse and human transcriptome information from various sources, including public domain and Celera ESTs, as well as genome-wide microarray data [47]. We found substantial expression in several dozens human and mouse tissues, but no indications for unusually high expression in testis. Our results support a neutral or near neutral evolution model for OR transcription control, whereby functionality is rendered less likely. All the results are fully available through HORDE [48].

## Results

### Collection of OR transcription data

Our first aim was to obtain evidence of transcription for human and mouse OR genes. We examined expression data from a multitude of sources, including cDNAs and mRNAs information as well as microarray data [47]. The source of microarray data is the GeneAtlas2 project, which provides whole genome expression patterns in dozens of human and mouse tissues [47].

A search through the GeneAtlas2 data identified (after filtration on potentially cross-reacting probesets) a total of 293 probesets matching 273 human ORs, and 397 probesets matching 371 mouse ORs. Probesets were then tested for being positively expressed applying the Cross-Gene Error Model on the full microarray data. We identified 206 human and 216 mouse OR genes as expressed in at least one tissue. This analysis included 61 human and 48 mouse tissues, among them mouse olfactory epithelium, mouse vomeronasal organ, and human and mouse olfactory bulb and testis. Every one of these tissues showed positive expression of at least one OR gene. Further information regarding expression of 221 human ORs and 587 mouse ORs was obtained from ESTs and mRNAs. This information was supported by 867 human transcripts in 78 tissues and 1965 mouse transcripts in 50 tissues.

**Figure 1:**
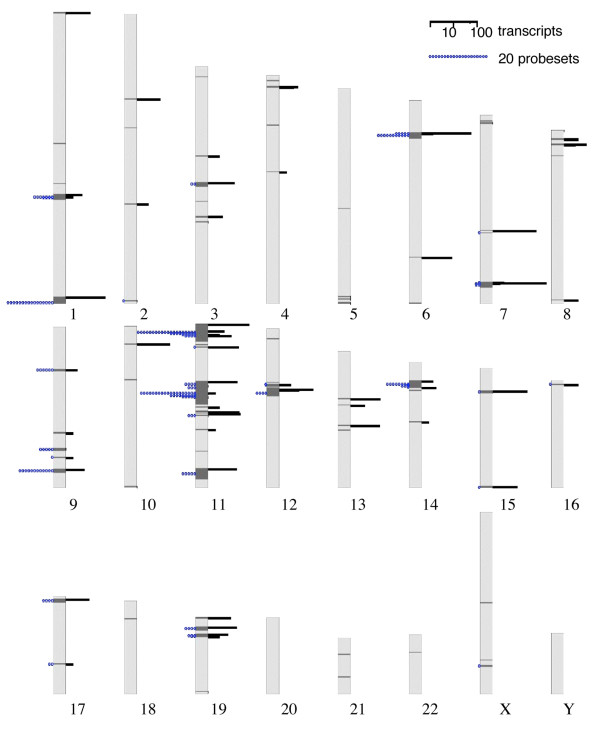
**Distribution of OR transcripts and probesets in the human genome**. A total of 867 transcripts and 206 probesets expressed in non-olfactory tissues, are shown in their approximate genomic location. OR clusters (as defined in HORDE [48]) are indicated on the chromosomes in dark grey rectangles with their width scaled to the number of ORs in the cluster. Nearby clusters may be merged. Black bars on the right specify the number of transcripts (ESTs or mRNAs) per cluster. Blue chains on the left represent the number of microarray expressed probesets in the cluster. Since distribution of clusters over chromosomes is not uniform, bars/chains are sometimes very close.

Altogether, we present evidence for the expression of 371 human ORs and 697 mouse ORs (see Additional file 1), which represent approximately 45% of both the human and mouse repertoires. Gene expression does not appear to be biased relative to particular genomic OR clusters (Figure 1), suggesting that an appreciable proportion of the entire genomic repertoire is likely to be transcribed.

### Ectopic expression of OR genes in non-olfactory tissues

**Figure 2:**
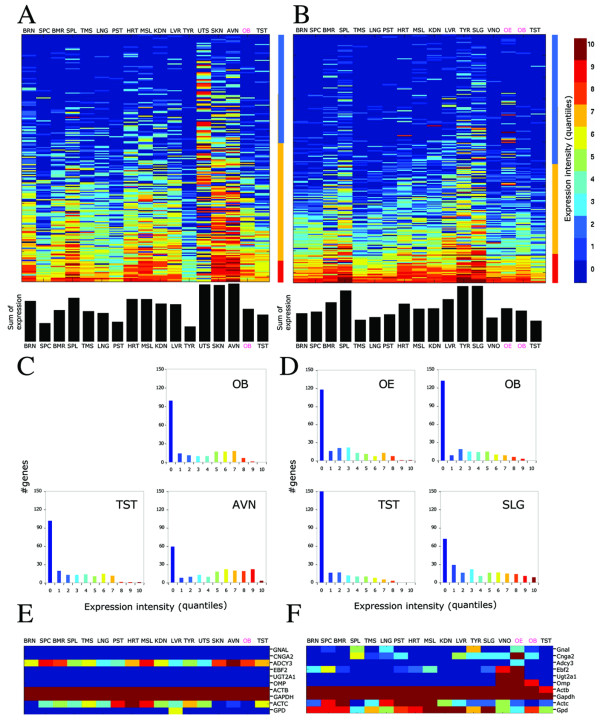
**Ectopic OR expression**. **A, B**. Expression profiles of OR genes are shown using intensity quantile scale, for 210 human probesets ORs (A) and 219 mouse probesets ORs (B). Quantile scale was defined using all probesets in a given tissue. The figure includes all GeneAtlas2 OR probesets that are unique and expressed in at least one tissue. Each row represents a probeset and each column represents a tissue. Probesets are sorted by the sum of their expression levels across all tissues. Blue in the colored column at the right of each panel specifies tissue specific genes, yellow midrange and red ubiquitously expressed (housekeeping) genes. The bars under the panel show the summated expression for each tissue. The tissue abbreviations in magenta indicate olfactory tissues (see also below). **C, D**. The distributions of expression intensities using same probesets as above, for some tissues in human (C) and mouse (D). **E, F**. Expression levels (in quantiles) for human (E) and mouse (F) non-OR genes including six olfactory related genes, olfactory G-protein (*GNAL*), cyclic nucleotide gated channel (*CNGA2*), adenylyl cyclase III (*ADCY3*), transcription factor early B-cell factor 2 (*EBF2*), enzyme UDP glycosyltransferase type 2 A1 (*UGT2A1*) and olfactory marker protein (*OMP*), as well as two housekeeping genes, actin beta (*ACTB*), glyceraldehyde-3-phosphate dehydrogenase (*GAPDH*) and two additional genes with an established function, actin alpha, cardiac muscle (*ACTC*) and glycerol 3phosphate-dehydrogenase *(GPD*). No expression data were available for the human olfactory epithelium. Tissue abbreviations: BRN, Brain; SPC, Spinal cord; BMR, Bone marrow; SPL, Spleen; TMS, Thymus; LNG, Lung; PNC, Pancreas; PST, Prostate; HRT, Heart; MSL, Skeletal muscle; KDN, Kidney; LVR, Liver; TST, Testis; OB, Olfactory bulb; OE, Olfactory epithelium; VO, Vomeronasal organ; AVN, Atrioventricular node; TYR, thyroid; UTS, uterus; SKN, skin; SLG, salivary gland.

**Figure 3:**
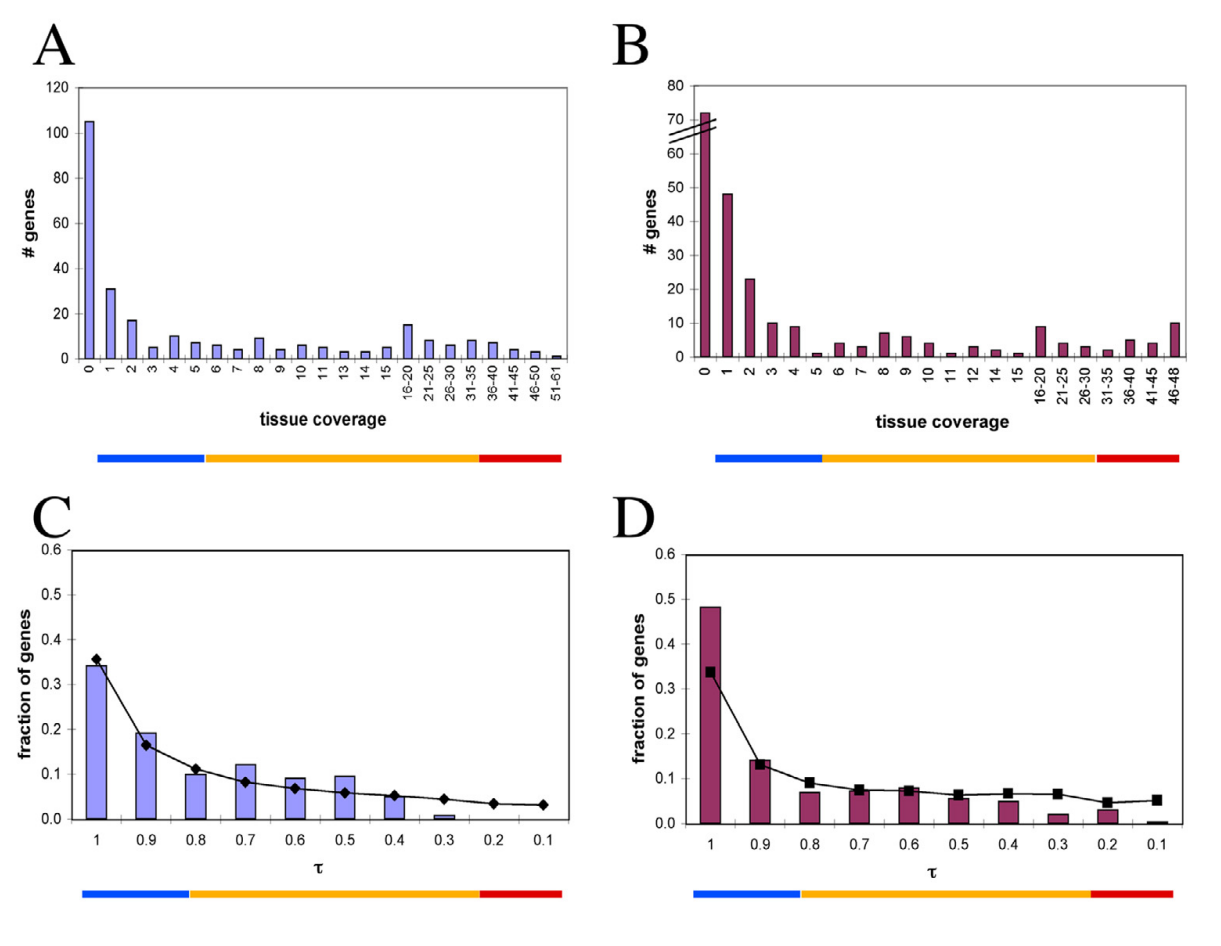
**Tissue specificity of the OR genes**. **A, B**. Distribution of the number of tissues in which genes are expressed for 210 human (A) and 219 mouse OR-related probesets from GeneAltas2. (B). **C**, **D**. The corresponding distributions for tissue specificity index (τ) values for human (C) and mouse (D), (bars). For comparison, the τ distribution of all the 15039 human and 13832 mouse gene profiles from the same source are superimposed (solid lines). The horizontal colored bars under the graphs show genes that are tissue specific (blue, left), midrange (yellow, middle) and ubiquitously expressed (red, right).

We examined the GeneAtlas2 data using a quantile-based scale [49] for all the expression values in the microarray. Ectopic OR gene expression was noticeable in a large diversity of non-olfactory tissues. We noted that in every tissue a group of ORs showed high expression level even when compared to all the genes included on the microarrays. The highest OR expression was observed in non-olfactory tissues, and these differed between human and mouse. In human, atrioventricular node, skin and uterus showed the highest quantile-based expression levels, while in the mouse such tissues were thyroid and salivary gland (Figure 2). While some ORs showed a relatively high specificity to particular tissues, others exhibited expression in a number of tissues (midrange genes [49]) and a few ORs had high quantile values in all or most tissues, i.e. behaved as housekeeping genes (Figure 2A,B and Figure 3). We examined the matrices of expression again after filtering out 36 human and 12 mouse OR-related probesets, whose specificity or sensitivity was less than 1. The general picture remained as before (see Additional file 2).

We asked whether the distribution of OR expression intensities was different in functionally implicated tissues as compared to other tissues. Generally, the distributions were rather similar for the two classes (Figure 2C,D). The functionally implicated tissues (olfactory epithelium and bulb, as well as testis) did not display an unusual distribution, nor did they show an unusually high number of highly expressed genes. The number of highly expressed ORs in testis was smaller than in olfactory epithelium and bulb (Figure 2C,D, red colors). Interestingly, these highly expressed ORs are not biased toward a genomic location or family affiliation [50]. In addition, highly expressed human and mouse ORs in the different tissues were not orthologous. More functionally related expression patterns were observed for a set of non-receptor genes with an established olfactory function (Figure 2E,F). Note that Olfactory Marker Protein (*OMP*) was expressed in mouse but not in human olfactory bulb. This might be the result of a poor probeset design since human *OMP *is not expressed in any tissue.

Across-tissue patterns of expression for individual OR genes were eclectic, differing widely from one gene to another (Figure 2A,B). A hierarchical clustering analysis was applied to 108 human and 141 mouse differentially expressed ORs, as determined using an ANOVA test applied to the GeneAtlas2 microarray data (see Additional file 3). The human OR gene tree ramified into two principal branches, whose major expression divergence was observed in the highly expressing tissues. In mouse, no significant clustering was observed. Principal component analysis also could not separate the ORs expression profiles into distinct groups (data not shown).

### Quantification of ectopic expression

**Figure 4:**
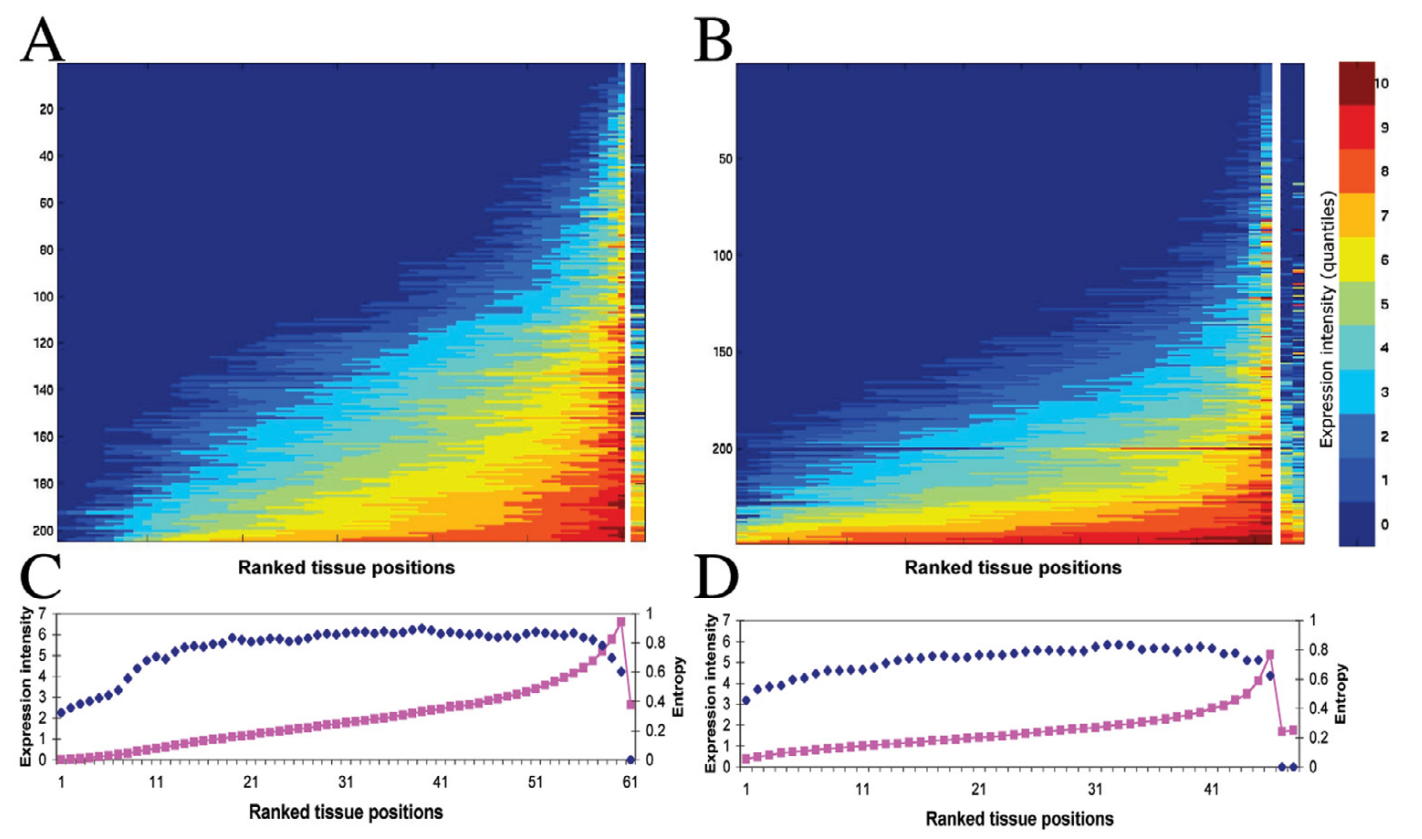
**Ranked quantification of OR ectopic expression**. **A, B**. Dually sorted matrices show ectopic expression for human (A) and mouse (B) OR genes. For every OR (rows) representing probesets were sorted according to the expression intensity, so the tissue with the highest expression level for that gene is on the right. The separate column(s) on the right represent the functional tissue(s): olfactory bulb in A and olfactory bulb (left) and epithelium (right) in B. The rows were sorted according to the row mean expression level (bottom is highest). Data included and expression color scale as in Figure 2. **C**, **D**. The mean expression level intensity (pink squares) and the entropy (blue diamonds) for each ranked position and for the functional tissue in human (C) and mouse (D).

To quantify ectopic expression, we ranked for each OR gene the ectopic tissues according to their expression level (Figure 4A,B), then for each ranked position we calculated the mean expression intensity and the entropy, which reflects the diversity of tissues in the given position (Figure 4C,D). The entropy was normalized to range from 0 to 1, where 0 indicates a single tissue population and 1 a population of all tissues. For OR genes, the entropy in most ranked positions was between 0.7 and 0.8, indicating that each position is populated with a great variety of tissues and that none of the tissues dominated the high expression positions, as would have been expected if ORs had a specific function in such tissues. Reanalyzing the data including olfactory tissues did not change the entropy values, an indication for the low level of ORs expression in the olfactory epithelium.

The product of the entropy and the expression intensity at each position was calculated and the mean of all products defined as the ectopic expression index, theoretically ranging from 0 (no ectopic expression) to 10 (all the examined genes are ubiquitously expressed and all the tissues are represented in every ranked position). The ectopic expression index was found to be 1.6 for human ORs and 1.3 for mouse ORs. Interestingly, a similar level of ectopic expression was observed in groups of brain specific genes (see Additional file 4) and spermatogenesis-related genes (see Additional file 5). In these gene groups, the entropy in most ectopic ranked tissue positions was above 0.7 and the ectopic expression index ranged from 1.1 and 1.5 for human and mouse brain specific genes respectively, to respectively 1.5 and 1.4 for spermatogenesis-related genes in human and mouse.

### Comparison with other microarray expression experiments

Recently, the results of a custom-made microarray, which includes all mouse OR genes hybridized to olfactory epithelium and 6 additional tissues (vomeronasal organ, lung, heart, testis, muscle and cerebellum) were published in Zhang et al. [17]. An examination of the 397 OR genes represented in both our analyzed experiment and that of Zhang et al., using the same cutoff criteria, revealed a significant concordance. Thus, all 48 ORs that showed olfactory epithelial expression in our analysis were also highly expressed in the results of Zhang et al. (see Additional file 6, p = 0.0001, chi-square test = 21).

**Figure 5:**
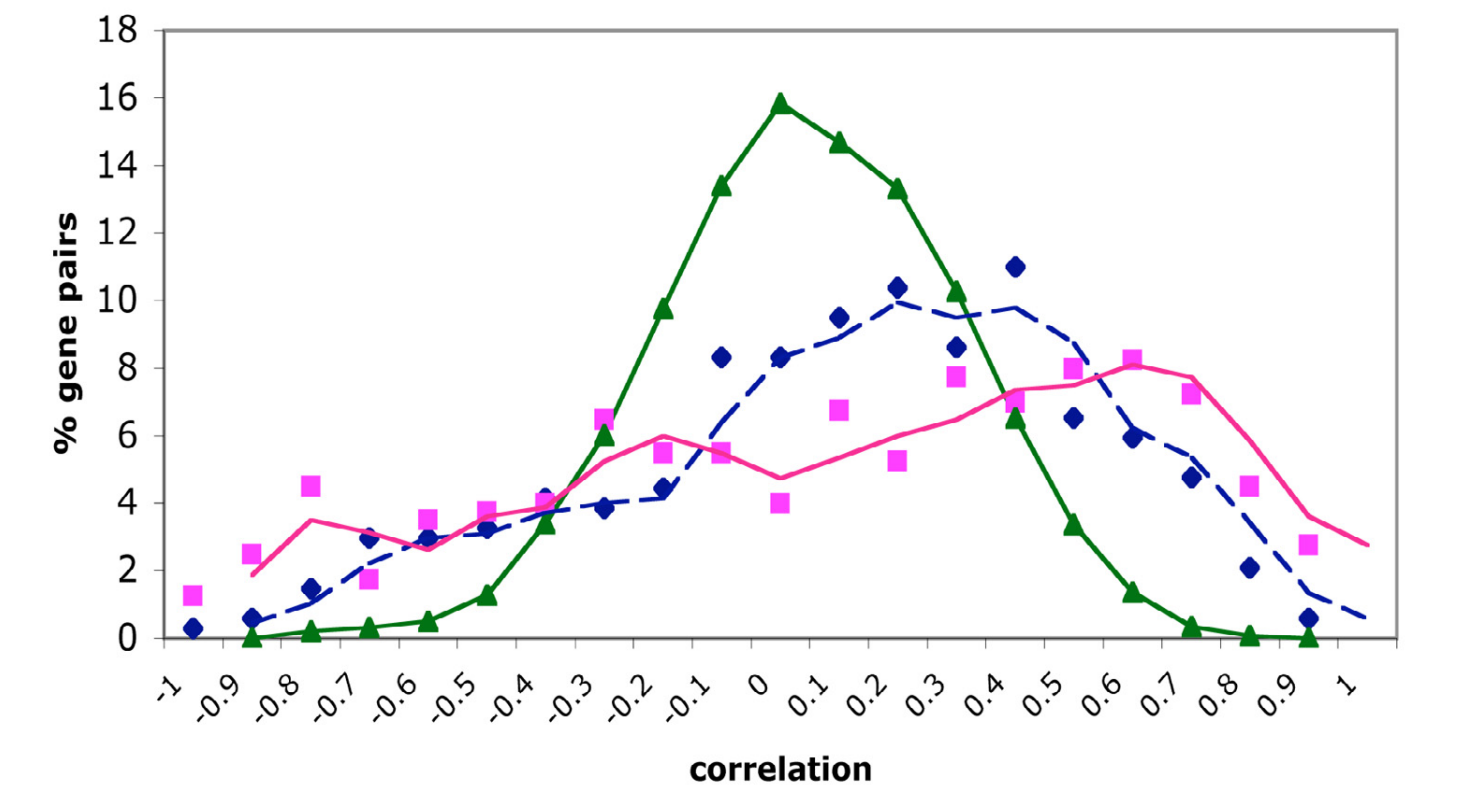
**Expression correlations between and within experiments**. Frequency distribution of Pearson's intragene correlations for probesets expression profiles of Zhang et al. [17] and Su et al. [47] data. Correlations were calculated for olfactory epithelium, vomeronasal organ, lung, heart, testis, muscle and cerebellum. Dashed (blue) line with diamonds represents the correlation values between 216 OR genes in Zhang et al. [17] and Su et al. [47] data. Solid (pink) line with squares within 402 probesets pairs from Zhang et al. [17], each pair representing the same OR in the coding region and in the 3' UTR. Solid (green) line with triangles for a set of 1000 randomly selected gene pairs probesets from Su et al. [47]. The first two lines were smoothed applying a moving average every 2 points. The first two correlation groups were not significantly different according to Wilcoxon test (p = 0.55), while these two differed from the third (Wilcoxon test, p < 0.0001).

We further compared the results of the two experiments by examining the correlation between expression profiles of genes that were represented in the two microarrays (Figure 5). The distribution of the correlation values was skewed towards positive values with a median of 0.17 and a most probable value of 0.4, results that show a highly significant deviation from randomness (Wilcoxon test, p < 0.0001). Interestingly, the distribution obtained from the correlation values of probesets extracted from the coding region versus the 3' untranslated region (UTR) of the same OR in Zhang et al. had a similar median (0.19), although the most probable correlation value was found to be higher: 0.6. We examined also correlations between expression profiles of two probesets representing the same OR gene in the GeneAtlas2 project. Only seven such probeset pairs were found and the mean correlation value between their expression profiles was 0.61 with a standard deviation of 0.13. Reexamination of Zhang et al. data revealed abundant ectopic expression not explicitly reported because of the stringent cutoff they used for defining differentially expressed genes.

### OR expression in sperm and testis

**Figure 6:**
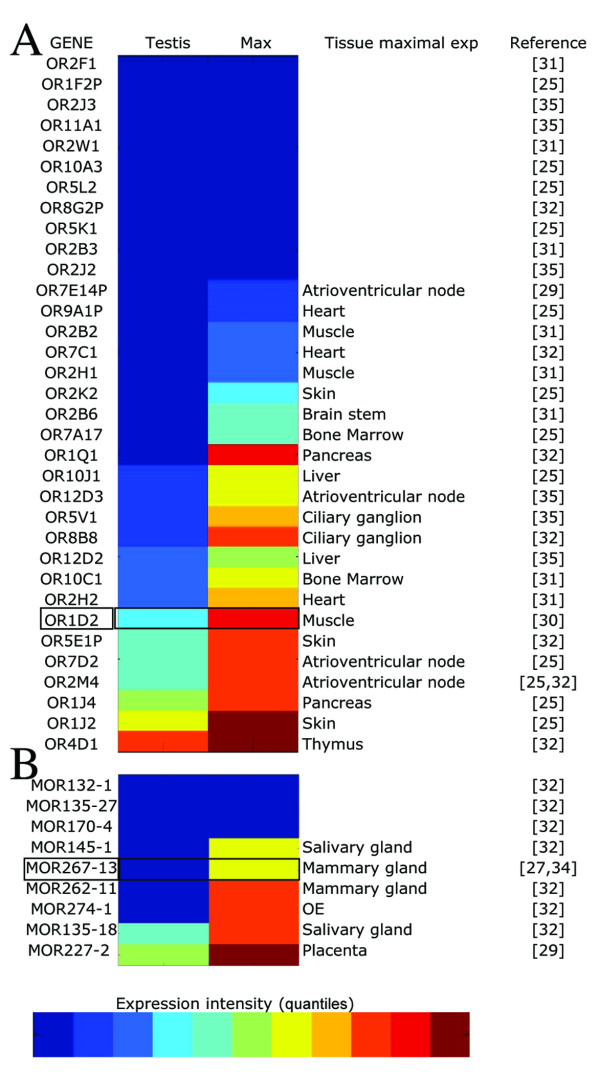
**Ectopic expression of OR genes in testis and in the tissue of maximal (Max) expression**. **A**. 34 human and **B**. 9 mouse ORs were represented in the GeneAtlas2 microarrays out of the 53 human and 19 mouse ORs previously reported to be expressed in testis or sperm. The OR gene symbols in black rectangles were suggested to be implicated in sperm chemotaxis. In the column right to Max is the name of the tissue with the maximum expression intensity, if expression was detected in any tissue in quantile 1 or higher. Expression color scale as in Figure 2.

Because of the presumed OR function in sperm chemotaxis, we examined human and mouse OR genes reported to be expressed in sperm and testis [26-31] and for which we had expression data. Most of these ORs were probably expressed in testis at levels below the noise-related detection threshold in these experiments (Figure 6A,B). In all cases where higher than background expression was detected, its level in testis was lower than in some other tissue(s). Two ORs, the human gene OR1D2 and the non-orthologous mouse gene MOR267-13 (outlined in black in Figure 6) have been the focus of more intense previous scrutiny in terms of a potential role in sperm chemotaxis towards the egg. Here, none of these genes showed their highest level of expression in testis nor did they show sperm/testis tissue specificity.

### Expression of OR genes and pseudogenes

**Figure 7:**
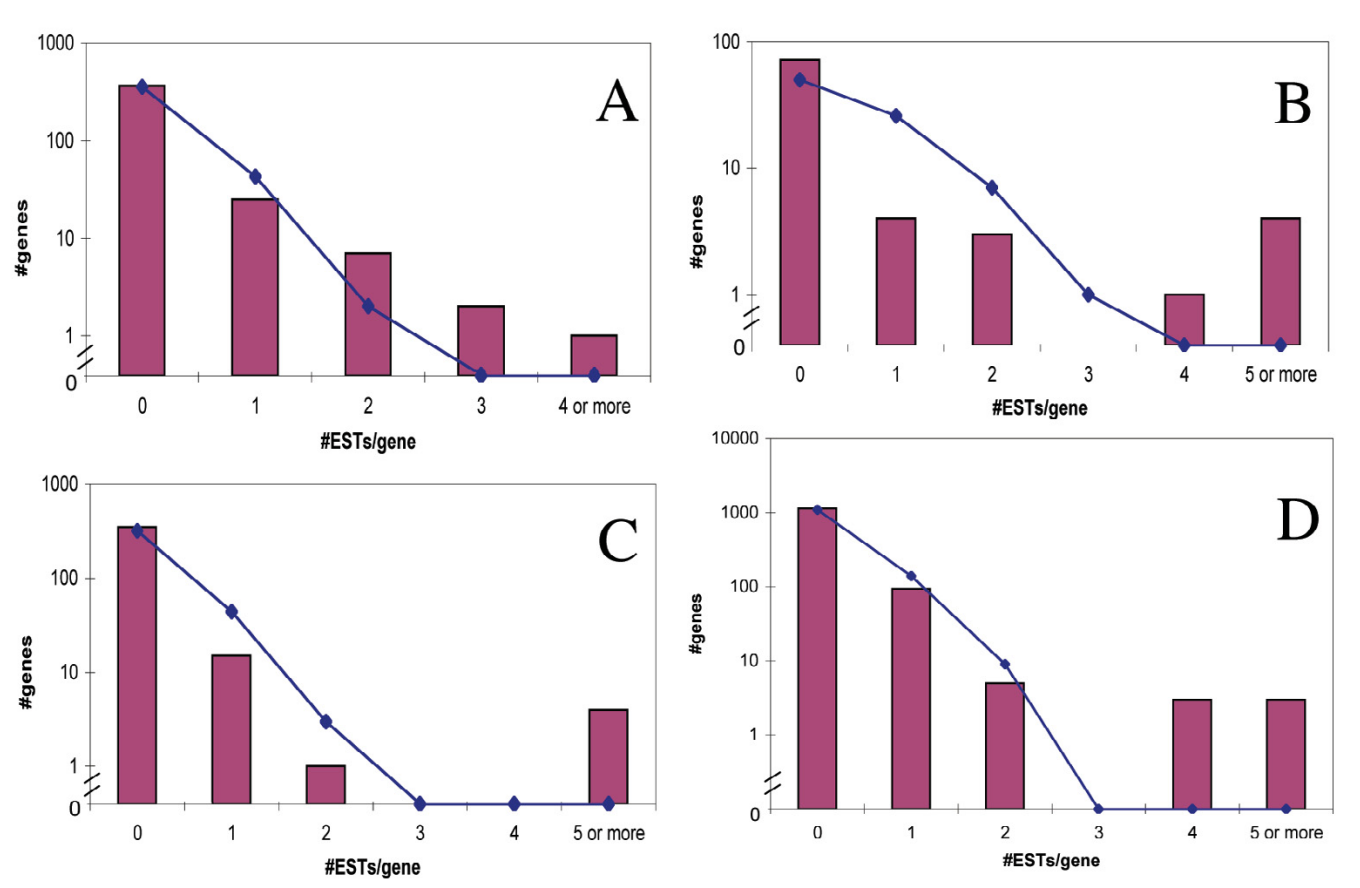
**Distribution of EST coverage for OR genes**. Bars show observed distributions for ESTs (spliced and non-spliced) coverage in non- olfactory tissues. The ESTs utilized for the distributions calculation were from normal tissues, non-subtracted and non-normalized libraries. Distributions for **A**. human intact ORs, **B**. human 7E subfamily ORs, **C**. human pseudogenes (excluding 7E subfamily members), **D**. mouse ORs (intact and pseudogenes). Solid lines are computed Poisson distributions based on the average value of ESTs per OR gene. The fit to the expected Poisson distributions was tested using the Kolmogorov-Smirnov test. The resulting p-values are: 0.998 for A, 0.003 for B, 0.401 for C and 0.591 for D.

We asked whether all ORs had an equal probability of being transcribed. For this purpose, we analyzed the ESTs data set, assumed to constitute a random representation of the transcribed OR repertoire. The distribution of ESTs per OR in human intact and pseudogenized genes (excluding pseudogenes belonging to subfamily 7E) was found to be in agreement with a Poisson distribution within statistical error (Figure 7A,B), as expected in case of equal transcription probability. A similar result was obtained for mouse ORs (Figure 7D). These results are somewhat tenuous because they could be affected by varying depth of EST sampling in different tissues. Reanalyzing the distributions based only on spliced ESTs did not change the agreement to Poisson distributions for intact and pseudogenized genes (excluding 7E members). Importantly, there was no significant bias against pseudogene transcription both in human and in mouse non-olfactory tissues (Table 1).

**Table 1:** Ectopic transcription coverage for intact and pseudogene OR genes in human and mouse. Transcripts include ESTs and mRNAs. Shown are the number and percentage (in parenthesis) of OR genes for which evidence of transcription was found, relative to the entire OR repertoire. Lowest row indicates the average EST coverage per OR, calculated using the count of adult normal tissues ESTs from non-normalized libraries in each subgroup. -7E indicates pseudogenes excluding 7E subfamily members. Percentage of OR genes for which evidence of transcription was found based only on spliced ESTs was 9% for intact, 8% for pseudogenized human ORs, and 5% for intact, 4% for pseudogenized mouse ORs. The EST coverage based only on spliced ESTs dropped to about 0.04 for intact genes and pseudogenes excluding 7E members, while coverage was 0.17 for the 7E subfamily, significantly different from that of the other groups.

	HUMAN		MOUSE	
	Intact	Pseudo	Intact	Pseudo
Transcripts	104 (26%)	117 (26%)	140 (14%)	45 (18%)
EST coverage	0.122	0.1360.529−7E7E︷0.204 MathType@MTEF@5@5@+=feaafiart1ev1aaatCvAUfKttLearuWrP9MDH5MBPbIqV92AaeXatLxBI9gBaebbnrfifHhDYfgasaacH8akY=wiFfYdH8Gipec8Eeeu0xXdbba9frFj0=OqFfea0dXdd9vqai=hGuQ8kuc9pgc9s8qqaq=dirpe0xb9q8qiLsFr0=vr0=vr0dc8meaabaqaciaacaGaaeqabaqabeGadaaakeaadaagbaqaauaabeqaciaaaeaacqaIWaamcqGGUaGlcqaIXaqmcqaIZaWmcqaI2aGnaeaacqaIWaamcqGGUaGlcqaI1aqncqaIYaGmcqaI5aqoaeaacqGHsislcqqG3aWncqqGfbqraeaacqqG3aWncqqGfbqraaaaleaacqaIWaamcqGGUaGlcqaIYaGmcqaIWaamcqaI0aanaOGaay5n+daaaa@41C9@	0.103	0.127

One OR subfamily, 7E, shows an unusually high average level of transcription, significantly different from the one of other intact and pseudogene ORs according to the Tukey-Kramer test (p < 0.05), (Table 1). Members of this subfamily were the only OR group for which the EST coverage differed significantly from a Poisson distribution (Figure 7B). In this skewed distribution, most 7E genes were under-expressed, while four genes, all belonging to one of the two 7E phylogenetic clades [51] were highly overexpressed. Most additional genes represented by ESTs belonged to the same over-expressed phylogenetic clade.

### Lack of correlation in orthologous OR expression profiles

**Figure 8:**
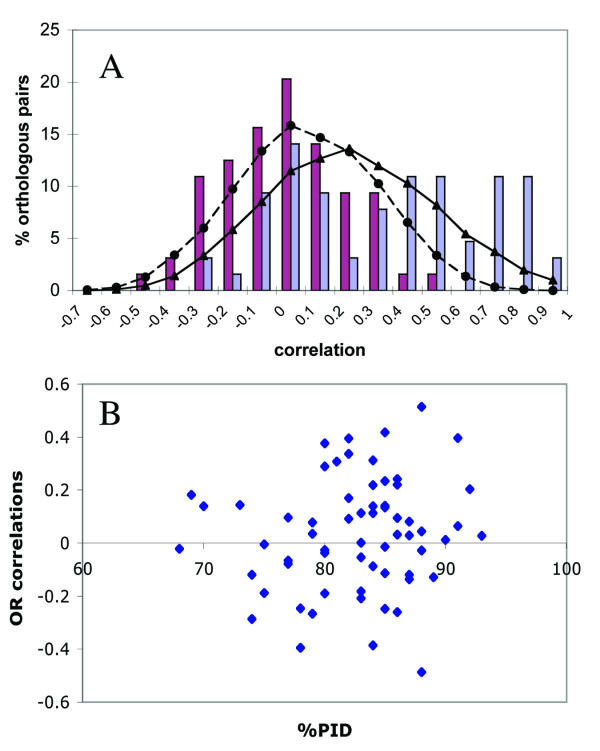
**Pearson's correlations between expression profiles of human and mouse orthologs**. Human and mouse expression profiles were extracted from the GeneAtlas2 project and the same methodology was used to normalize both of them. **A**. Frequency distributions of Pearson's correlations for a set of 9616 mouse-human orthologs (solid line with triangles) and a set of 9616 mouse-human randomly chosen gene pairs (dashed line with circles) as well as for 64 mouse-human orthologous OR pairs (dark gray/pink bars) and 64 mouse-human spermatogenesis related orthologous genes (light gray/blue bars). A significant difference between the correlations values in the orthologous OR and spermatogenesis genes groups was supported by Wilcoxon test (p < 0.0001). **B**. Pearson's correlations between mouse-human orthologous OR pairs were plotted against the protein identity percent within each pair.

Out of 122 orthologous pairs of OR genes as previously defined [13], 64 OR pairs were found to be represented in both the human and mouse GeneAtlas2 microarrays [47]. This allowed us to examine the relationship between their expression profiles in the 20 normal tissues shared by the reported human and mouse data (Figure 8A). Pearson correlations between the expression profiles of each pair were normally distributed around a mean of zero, suggesting that no significant correlation existed between the orthologous expression profiles. A nearly identical distribution was seen for random pairs. In addition, the Pearson correlations were independent of the protein sequence divergence between orthologs (Figure 8B). No significant correlation was observed when human and mouse orthologs were analyzed for each tissue separately (not shown), including the functionally implicated olfactory bulb and testis where ortholog correlation is more likely to be observed [52]. In a control test, we compared a set of all 9616 human-mouse orthologous genes present on the arrays, as well as 64 human-mouse orthologous pairs of genes participating in spermatogenesis. The distribution for the former had a mean of 0.15, in agreement with previous reports comparing orthologs using the same platform and analysis methods [52,53] and significantly different from the random pairs distribution (t-test, p < 10^-16^). For the spermatogenesis related genes the mean was 0.38 (Figure 8A) with a positive Pearson correlation in testis (r = 0.65, p < 10^-16^). It is important to note that all the experiments compared here were performed in the same laboratory using the same platform and analysis methods, thus variations in experimental parameters between species could not account for the observed lack of correlation between OR orthologs.

### Relationship between OR expression and genomic neighborhood

We examined the possibility that OR gene expression is related to genomic location, and potentially influenced by the transcription of non-OR neighboring genes, namely genes not belonging to the OR superfamily and located at a maximum distance of 0.5 M from an OR. We found that 98 out of 165 human ORs (59%) represented by at least one EST whose source was non-olfactory tissues, have a non-OR neighbor. This is in contrast to 241 out of 684 genes (35%) for ORs not observed to be ectopically expressed (p < 0.0001, chi-square test = 36). In mouse, a similar but weaker trend was found: 51% (80 out of 156 genes) of the ectopically expressed ORs had a non-OR genomic neighbor while the fraction was 42% (317 out of 749 genes) for ORs not observed to be ectopically expressed (p= 0.04, chi-square test = 4.21).

## Discussion

### Wide range of ectopic OR transcription

Motivated by the need to provide evidence of OR transcription, we searched through numerous databases for transcription evidence and successfully provided such information for 371 human OR genes. Since the nominal expression site of OR genes, namely olfactory epithelium, is practically not represented in transcript databases, most of the expression evidence presented here originates from ectopic tissues. Our results generally indicate that hundreds of OR genes have active promoters capable of directing transcription in diverse cellular contexts. As the samples explored are basically unbiased, it is suggested that our results are representative of the entire olfactory receptor gene repertoire.

It could be argued that the ectopic OR expression is an artifact of microarray experiments. Indeed, microarray experiments are known to be "noisy" [54], requiring independent experimental validation. Such validation is presently underway for selected OR genes that show unique patterns of expression, and preliminary results show adequate concordance for two genes in terms of tissue-average expression levels, though not yet for individual tissue levels. However, the essence of the presently reported results, namely the widespread occurrence of OR expression in ectopic tissues is amply supported by previous reports showing ectopic OR transcription by PCR and sequencing methods [25,29,36,37,55]. The identification of numerous OR-related ESTs in diverse tissue origins provides further corroboration. Moreover, since we carefully selected the OR probesets to avoid possible cross-reaction between similar ORs, the observed expression levels could not be a result of summation over numerous genes, each expressed in negligible amounts. In fact, some of the OR genes were found to be highly expressed well above the background level.

Ectopic expression is a universal phenomenon that extends beyond the OR gene superfamily. Here, we corroborate this phenomenon for two additional gene groups, brain specific and spermatogenesis-related genes. Ectopic expression has been also observed previously at the protein level in enzymes and neurotransmitters and it has been suggested to be widespread in the cells of multicellular organisms [56].

### Comparison between the olfactory epithelium and other tissues

Our findings do not indicate unusually high OR expression levels in olfactory epithelium relative to other tissues. This expression is specific for mature olfactory sensory neurons and not for other cell types in the olfactory epithelium, as previously reported [57,58]. At face value, such result could be interpreted as contradicting a function for OR genes in olfactory epithelium. However, as each OR gene is expressed only in approximately 0.1% of the sensory neurons, OR transcripts are diluted in the total olfactory tissue mRNA.

We compared the results of our analyses to the ones of an expression custom-made microarray published by Zhang et al. [17]. We identified in the GeneAtlas2 microarray 397 mouse unique OR-related probesets representing 371 genes, we could not detect expression in any tissue for 181 probesets (155 genes), from the remaining 216 OR genes, 117 were not found to be expressed in the olfactory epithelium and only 32 were expressed in the five upper quantiles. In contrast to our results, they found a higher fraction of OR genes that surpass the threshold and are called "enriched in the olfactory epithelium" as compared to fraction of expressed in the olfactory epithelium as emerging from the data analyzed here. Su et al. [47] used 5 μg of cRNA in the microarray hybridization experiments, while Zhang et al. used 70 μg. This difference is a possible explanation as to why we fail to identify olfactory epithelium-specific OR transcription.

The median correlation coefficient between the two experiments is in line with those in other reports, ranging from 0.2 to 0.4 in comparisons among datasets acquired for identical RNA samples but in different laboratories and using different experimental and analysis protocols [54]. Other factors which may account for the differences in the two experiments include the use of mRNA from different mouse strains, differences in probeset designs and differences in data processing software. The influence of the probeset design is critical, as learned from comparisons of different results in the same report (Zhang et al.).

### OR genes in testis

It is widely believed that the main function of OR proteins is in mediating olfaction within the chemosensory organ. In parallel, reports published ever since the discovery of OR genes have proposed an additional OR function, namely as mediators of sperm chemotaxis. In the past decade, considerable evidence has accumulated in support for this hypothesis [25-31], but the concept has also been disputed [59]. The present study, which addresses the expression of a large number of OR genes in olfactory tissues as well as in other tissues, including testis, could help to shed light on this issue. One of the important questions is which of the hundreds of OR genes is actually involved in sperm chemotaxis, as such specific function is unlikely to be mediated by the entire OR repertoire. Previous functional studies have focused on two OR genes: the human OR1D2 (hOR17-4) [33] and a non-orthologous mouse gene, MOR267-13 (MOR23) [34]. For MOR267-13 it was stated that it is expressed at low level in testis [34], this is also supported by our results. We found that also OR1D2 is expressed at low level in testis. Our results further show that these two specific ORs have a higher expression in other tissues. Conversely, other ORs are highly expressed in testis but have not been invoked so far as chemotaxis mediators. It should be noted, however, that genes could function in an ectopic tissue (e.g. sperm) even if they are expressed at higher level in another tissue. The notion that a gene must be expressed at the highest level in the tissue in which it is functional is plausible but exceptions may exist.

### OR ectopic expression may be selectively neutral

In addition to a proposed role in sperm chemotaxis, ORs have been proposed specific roles in numerous other tissues, mostly based on the mere presence of OR transcript(s). This includes cell-cell recognition and organ construction during development [37,41,45,46], taste perception [36], chemical detection of exogenous or endogenous ligands in the cerebral cortex [45] and additional functions [40,43,44]. Does OR expression in a given ectopic tissue indicate specific functionality? An alternative scenario would be that ectopic OR transcription is predominantly the result of neutral or nearly neutral mechanisms, e.g. small DNA sequence changes in regulatory regions, fixed in the population by random drift and not necessarily related to function or fitness [52,60,61].

A claim that ectopic OR expression may be in part governed by neutral changes is supported by several findings. First, the divergence in the expression patterns of human-mouse orthologous OR pairs in ectopic tissues is similar to that of randomly selected pairs. This observation generalizes a previously reported specific case for OR51E2, which was found to be over-expressed in human prostate [39,40], although minor expression could be detected also in brain. Its mouse ortholog was found predominantly in brain, and its rat ortholog in brain and liver [43]. Second, OR transcription was found here to be unbiased with respect to any particular OR subgroup in ectopic tissues (except for the human 7E subfamily members, see below), as indicated by the approximate fit to a Poisson distribution. We note, though, that other studies have reported up to 300-fold difference in the levels of ORs transcription in mouse olfactory epithelium [5,16]. Last, OR pseudogenes were found here to be expressed in ectopic tissues at similar levels to those of intact genes. In contrast, in olfactory epithelium, where chemosensory functionality is expected, OR pseudogenes were found to be significantly less expressed than intact genes [16]. In spite of this, our results can neither exclude nor confirm the possibility that some ORs do have a function in ectopic tissues, as the functionally-related transcription might be concealed within the relatively noisy microarray data.

The relative weight of neutral evolution and purifying selection in shaping gene expression changes is controversial. Based on the expression data that have accumulated lately, a predominantly neutral model of evolution for gene expression has been proposed [52,60,61]. On the other hand, others [62] have recently indicated the parallel importance of selective constraints. The neutral model was previously supported by a lack of correlation between gene expression profiles and gene sequence divergence [52], a general observation that is in line with our own results. Also, similar rates of divergence in gene expression levels between humans and chimpanzees were found for intact and expressed pseudogenes [60], again echoing the results reported here.

Recently, ectopic expression and its evolutionary significance have been reviewed [56]. The authors propose that transcription regulation is leaky due to the need to decondense chromatin and the limited number of transcription factors in the cell. They suggest that ectopic expression, which appears randomly, may have evolutionary potential and provide an opportunity to develop protein function diversification ("the marginal benefit hypothesis"). The same might be true for OR genes, a point that will have to be further explored.

### The 7E subfamily

The OR gene subfamily 7E has expanded extensively in the primate lineage, composing ~10% of the human and chimpanzee OR repertoires. In humans, all its members but one are pseudogenes [51,63]. It was suggested that the subfamily expansion occurred through a complex mechanism of large segmental duplications, and that the duplication unit included one 7E subfamily member from each of the two phylogenetic clades of the subfamily [51]. Our results suggest that one of these two genes had already a disrupted promoter when the expansion started. The unusually high expression level of the second clade may be the result of mutations enhancing expression. It is also possible that highly expressed 7E members have some potential function after all as previously suggested [51].

### OR genomic neighborhood

Previous reports have shown that adjacent and nearby genes show correlated expression patterns in yeast [64]. Human genes were shown not to be randomly distributed in the genome; highly expressed genes and weakly expressed genes tend to populate different chromosomal domains [65,66]. It has been suggested that co-expressed genes in eukaryotic genomes reflect the domain organization of chromatin [67]. We have noticed that ORs located within the range of 0.5 M from non-OR genes have a higher tendency to be expressed than others, hinting that the genomic environment may partially influence OR gene expression. This is further supported by the expression of an OR gene in erythroid cells, attributed to its genomic location in the transcriptionally active chromatin domain of the extended β-globin gene cluster [38].

## Conclusion

We reported on widespread ectopic OR expression. Related observations have formed the basis for proposing specific roles for ORs in non-olfactory tissues. We proposed an additional explanation for ectopic expression, a neutral or near neutral model of evolution for at least part of OR transcription regulation in non-olfactory tissues. This is supported by the uniform overall transcription level in most tissues, the heterogeneity in its patterns, the expression level proportion similarity in OR intact and disrupted genes and the total lack of correlation between human and mouse orthologous ORs.

## Methods

### Identification of OR genes in GeneAtlas2 microarrays

Human OR probesets were identified based on genomic location overlap between GeneAtlas2 targets and HORDE annotation [48]. Mouse OR probeset identity was established using the UCSC genome annotation database table knownToGnf1m.txt.gz [68], which connects between probesets and GenBank accession numbers. Gene symbols and definitions were assigned to the accession number using Ensembl annotations downloaded via EnsMart [69].

We validated the probesets annotation using BLAT [70]. A probe was matched to an OR gene if it aligned in the correct orientation, with no more than one mismatch, and did not align to any additional OR gene or to any other gene from Ensemble all human/mouse transcripts library. Following the GeneAnnot algorithm described in [71], sensitivity and specificity scores were calculated for each probeset. Sensitivity score describes the percentage of probes in a probeset that match a gene and specificity score denotes how many other genes match the probeset and to how many probes within the probeset they match. Probesets having sensitivity or specificity lower than 0.7 were excluded and not used on further analyses. After filtering the probesets we were left with representation for 273 human and 371 mouse ORs (293 human and 397 mouse probesets). Most remaining probesets were of high quality, with both sensitivity and specificity scores of 1 (79% in human and 91 % in mouse), (see Additional file 7).

### Scaling GeneAtlas2 expression level intensities

Expression data for human and mouse tissues were obtained from GNF Symatlas [72]. The human and mouse arrays data were supplied after applying the MAS5 (Affymetrix, Santa Clara, CA) algorithm and normalization using global median scaling [47]. Sixty one human normal adult tissues and 48 mouse normal adult tissues were selected for the analysis.

Log_10 _of the expression values were averaged for replicas and were divided into 11 bins according to [49]. Intensities lower than log_10_200 for mouse and log_10_300 for human were considered as the zero bin. The remaining intensities were divided into 10 equal density quantiles.

Throughout this work quantile scaling was utilized, unless otherwise stated.

### Tissue specificity index calculation

A graded tissue specificity index, τ, ranging from 0 (ubiquitously expressed) to 1 (one-tissue specific) was calculated as described in Yanai et al. [49].

### GeneSpring analyses

Expression data was analyzed also applying GeneSpring software. This was used for determining the number of positively expressed genes, differentially expressed genes, and clustering. Each expression value was divided by the median of the microarray and the median of the probeset across all tissues. Unexpressed probesets were filtered out using the Cross-Gene Error Model. Differentially expressed genes were determined by an ANOVA test, p-value of 0.001. Under these conditions, 206 human and 216 mouse OR genes were found expressed, and 108 human and 141 mouse ORs were differentially expressed. Hierarchical gene clustering (gene tree) and tissue clustering (condition tree) were performed for differentially expressed ORs.

To verify that expression data from all the tissues have the same distribution (normal), we calculated the distribution of the expression log values for each tissue and its skewness parameter. Tissues with a skewness parameter outside the range of average plus 2 standard deviations of all skewness values were removed from further analyses. The removed tissues were pancreas, brown fat and tongue in mouse and cervical ganglion, trigeminal ganglion, ovary and appendix in human.

### Ectopic expression quantification

To quantify ectopic expression, we ranked for each OR gene the ectopic tissues according to their expression level, entropy and mean expression were then calculated for each ranked position. The Shannon entropy (H) formula [73] was utilized to measure the entropy:

H=−∑i=1TPi log2Pi
 MathType@MTEF@5@5@+=feaafiart1ev1aaatCvAUfKttLearuWrP9MDH5MBPbIqV92AaeXatLxBI9gBaebbnrfifHhDYfgasaacH8akY=wiFfYdH8Gipec8Eeeu0xXdbba9frFj0=OqFfea0dXdd9vqai=hGuQ8kuc9pgc9s8qqaq=dirpe0xb9q8qiLsFr0=vr0=vr0dc8meaabaqaciaacaGaaeqabaqabeGadaaakeaacqqGibascqGH9aqpcqGHsisldaaeWbqaaiabbcfaqjabbMgaPjaaykW7cqqGSbaBcqqGVbWBcqqGNbWzdaWgaaWcbaGaeGOmaidabeaakiabbcfaqjabbMgaPbWcbaGaeeyAaKMaeyypa0JaeGymaedabaGaeeivaqfaniabggHiLdaaaa@4242@

where Pi is the fraction of the tissue type i, and T is the total number of tissues. The entropy was normalized dividing by log_2 _T, to range from 0 to 1.

### Choice of brain-specific and spermatogenesis gene groups

Brain specific genes were selected based on scientific literature. We search Pubmed [74] for papers including the exact phrases "*brain specific*" or "*brain-specific*" in the title or abstract. The list of genes was then manually extracted and carefully curated by inspection and reading of the title/abstract. Probesets/genes assignment was based on the GNF organization annotation [75]. The final list included 57 human brain specific genes and 50 mouse brain specific genes. Human spermatogenesis related genes were identified searching with the keyword "*spermatogenesis*" in GeneAtlas web site. Corresponding mouse orthologs for the two groups were extracted from GeneCards [76].

### Human mouse comparisons

Human-mouse OR orthologs identification was based on a previous work, which identified human-mouse-dog three way mutual best hits [13]. GeneCards was further used to extract 9616 human-mouse orthologous pairs represented in the mouse and human microarrays. Random pairs were selected from this list using the PERL rand function. Pearson correlations were calculated using the log_10 _expression values supplied by Su et al., since these values are normally distributed.

### ESTs and mRNA data mining and procedures

Data mining of ESTs and mRNA was performed using UCSC genome browser annotation tables chr#_est.txt.gz and chr#_mrna.txt.gz, (# represents the chromosome number) [68]. We selected ESTs and mRNAs which were aligned to ORs coding regions.

After curation (see below), ESTs and mRNAs that included part of the UTR, were used as probes for extracting additional ESTs (in two data mining rounds). In addition, spliced ESTs which were aligned upstream of the coding region (up to 10 Kb) were collected. ESTs and mRNAs annotations regarding tissue source and type (tumor or normal) were extracted from GenBank and MEROPS [77].

Data mining from Celera Genomics [78] was performed using all OR coding sequences as queries in a BLASTN search against Full Invitrogen sequences (FIS) and 5' Invitrogen clones libraries. Hits with an e-value of less than 10^-10 ^were downloaded to our computer. The final assignment of a particular Celera's cDNAs to a particular OR was based on an alignment >97% over 500 bp.

### ESTs and mRNA curation

ESTs were rechecked to represent OR genes by excluding those whose genomic locations overlapped exons of the non-OR subset from the UCSC known gene table [68]. Spliced ESTs were required to be transcribed from the same strand as the OR. This criterion was not applied to non-spliced ESTs, because their transcription strand is less reliable since they lack splice junctions that verify the strand. We applied a cutoff of maximum 500 bp length for non coding exons and maximum distance of 100 Kb between a non coding exon and the coding region. The first criterion was based on previous knowledge about the typical OR UTR structure [18,31] and was applied to avoid genomic contamination, the second was applied to avoid collection of chimeras (transcripts including very large introns are suspected to be chimeras). Redundant information from ESTs that belong to the same clone was removed. mRNAs which their GenBank definition matched non-OR genes were manually removed from the dataset.

The final dataset contained 867 human transcripts, including 721 ESTs (spliced and non-spliced) and 146 mRNAs, from 78 tissues. These provided evidence for transcription of 221 human ORs. In mouse we obtained 1860 ESTs and 105 mRNAs, from 50 tissues. Of these 1176 ESTs are the result of a single project [16]. Together they provided transcription evidence for 587 mouse ORs.

### ESTs distribution analysis

Poisson distributions were calculated using ESTs from the first data mining round, as these constitute a true random set. Also, we filtered out ESTs whose source was not normal adult tissue as well as ESTs from normalized or subtracted libraries. Kolmogorov-Sminorv tests were performed at the web server [79] or using the Matlab6p5 application software.

## Authors' contributions

EF performed data mining, conducted the statistical analyses and drafted the manuscript. TO participated in the data mining, performed the 7E subfamily analyses and drafted the manuscript. MK carried out experimental work. RO participated in the comparison between microarray experiments and helped with the GeneSpring software. IY participated in the data mining. DL participated in the study design and revised the manuscript. All authors read and approved the final manuscript.

## Supplementary Material

Additional File 1 A list of the ORs found to be expressed is provided in Additional file 1 Click here for file

Additional File 2 A figure showing ectopic OR expression including unique probesets whose specificity and sensitivity are 1 is shown in Additional file 2 Click here for file

Additional File 3 The hierarchical clustering of differentially expressed ORs is in Additional file 3 Click here for file

Additional File 4 Figures showing ectopic expression of brain specific and spermatogenesis related genes across 61 human tissues are shown in Additional file 4 Click here for file

Additional File 5 Figures showing ectopic expression of brain specific and spermatogenesis related genes across 48 mouse tissues are shown in Additional file 5 Click here for file

Additional File 6 A comparison between expected and observed combinations of expressed and non-expressed OR genes in the experiments of Su et al. and Zhang et al., is available in Additional file 6 Click here for file

Additional File 7 The sensitivity and specificity scores for the probesets representing OR genes are available in Additional file 7 Click here for file
