# Supplementary material for: Widespread ectopic expression of olfactory receptor genes
Source: BMC Genomics. 2006 May 22;7:121. doi: 10.1186/1471-2164-7-121 (PMC1508154; doi:10.1186/1471-2164-7-121)
Supplement: Additional File 2 — A figure showing ectopic OR expression including unique probesets whose specificity and sensitivity are 1 is shown in Additional file 2 [file 1471-2164-7-121-S2.pdf]

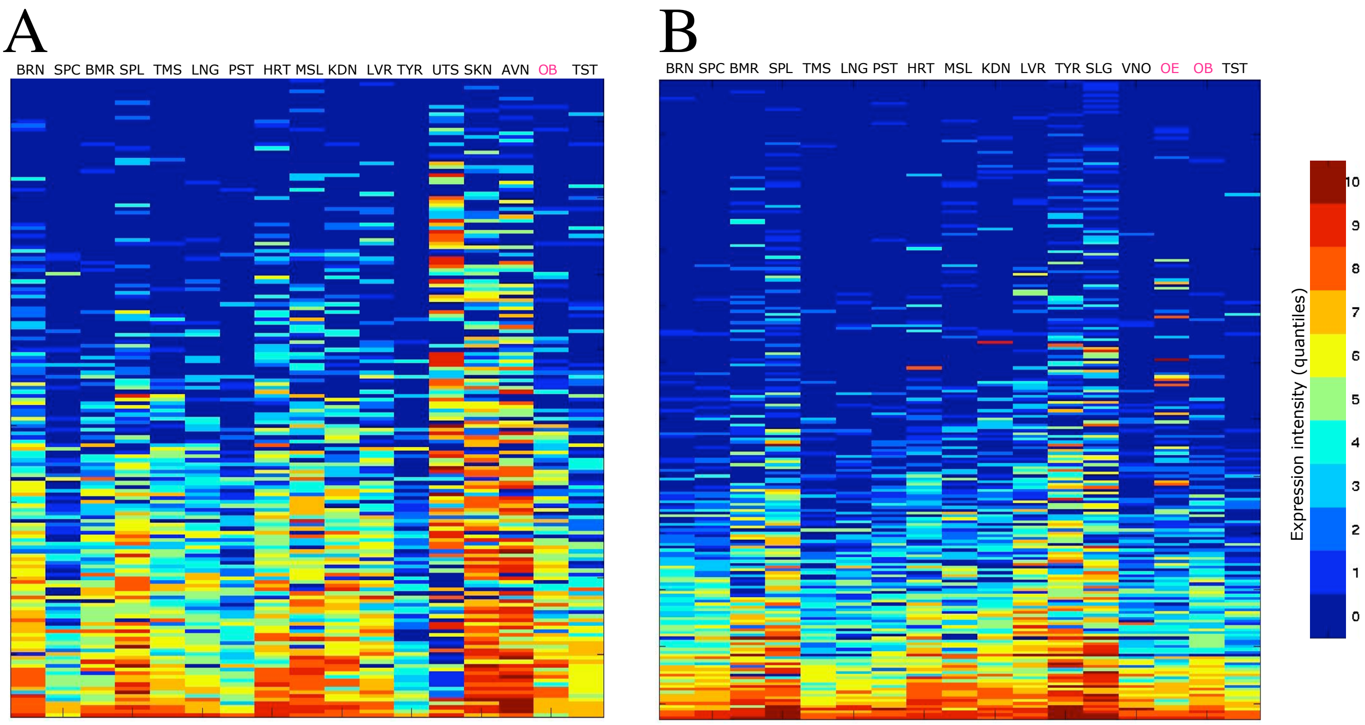

Ectopic OR expression. A, B. Expression profiles of OR genes are shown using intensity quantile scale, for 174 human probesets (A) and 207 mouse probesets (B). Quantile scale was defined using all probesets in a given tissue. The figure includes all GeneAtlas2 OR probesets whose specificity and sensitivity are equal to one and are expressed in at least one tissue. Each row represents a probeset and each column represents a tissue. Probesets are sorted by the sum of their expression levels across all tissues. Olfactory tissues abbreviations are in magenta. Tissue abbreviations: BRN, Brain; SPC, Spinal cord; BMR, Bone marrow; SPL, Spleen; TMS, Thymus; LNG, Lung; PNC, Pancreas; PST, Prostate; HRT, Heart; MSL, Skeletal muscle; KDN, Kidney; LVR, Liver; TST, Testis; OB, Olfactory bulb; OE, Olfactory epithelium; VO, Vomeronasal organ; AVN, Atrioventricular node; TYR, thyroid; UTS, uterus; SKN, skin; SLG, salivary gland.
