# Supplementary material for: Widespread ectopic expression of olfactory receptor genes
Source: BMC Genomics. 2006 May 22;7:121. doi: 10.1186/1471-2164-7-121 (PMC1508154; doi:10.1186/1471-2164-7-121)
Supplement: Additional File 3 — The hierarchical clustering of differentially expressed ORs is in Additional file 3 [file 1471-2164-7-121-S3.pdf]

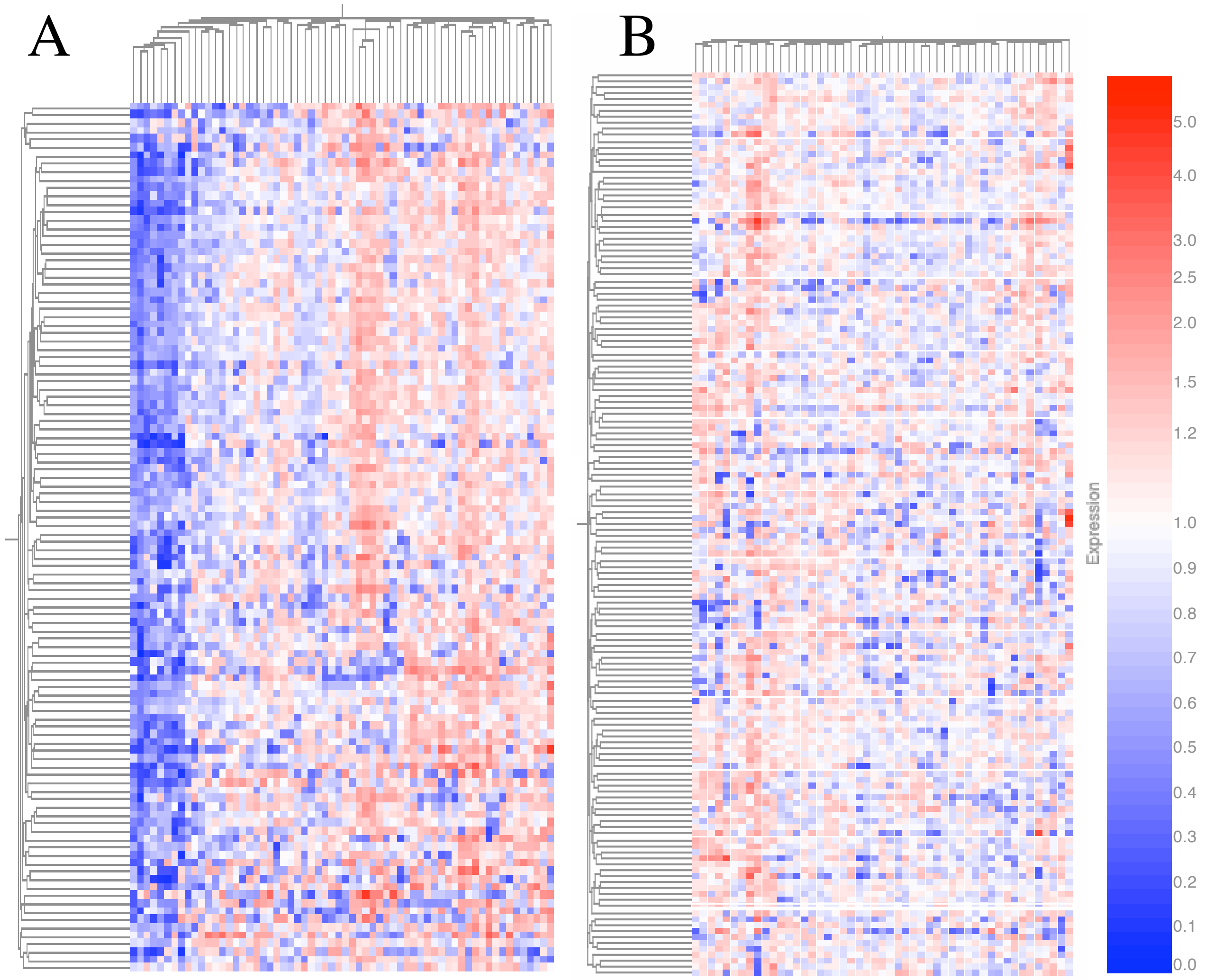

Expression profiles of GeneAtlas2 OR-related probesets. A, B. Hierarchical clustering of differentially expressed probesets for108 human ORs in 61 tissues (A) and 141 mouse ORs in 48 tissues (B). Each row represents a probeset and each column represents a tissue.
