# Supplementary material for: Widespread ectopic expression of olfactory receptor genes
Source: BMC Genomics. 2006 May 22;7:121. doi: 10.1186/1471-2164-7-121 (PMC1508154; doi:10.1186/1471-2164-7-121)
Supplement: Additional File 4 — Figures showing ectopic expression of brain specific and spermatogenesis related genes across 61 human tissues are shown in Additional file 4 [file 1471-2164-7-121-S4.pdf]

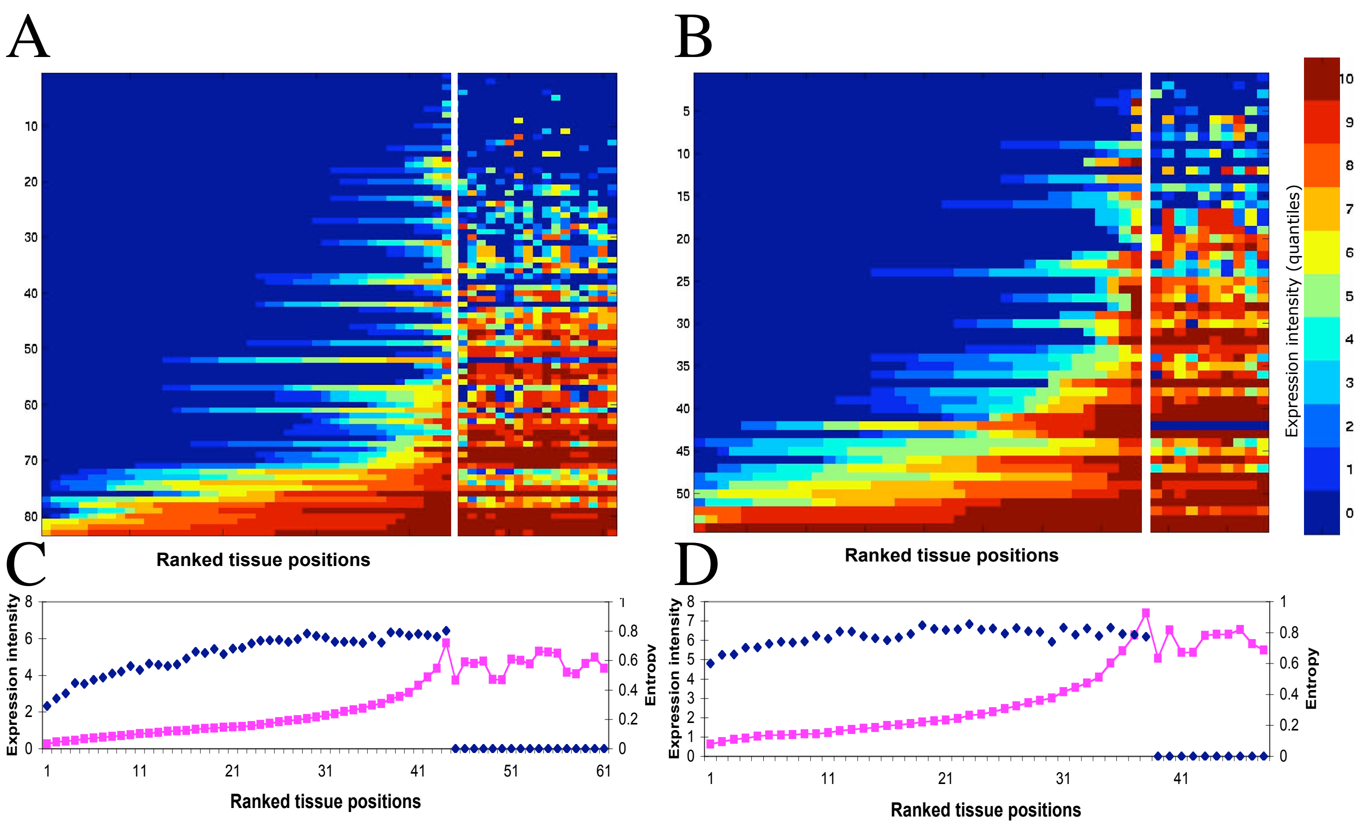

Ranked quantification of brain specific genes ectopic expression. A, B. Dually sorted matrices show ectopic expression for human (A) and mouse (B) brain specific genes. For every gene (rows) representing probesets were sorted according to the expression intensity, so the tissue with the highest expression level for that gene is on the right. The separate columns on the right represent the functional tissues, whole brain and different parts of it including temporal lobe, globus pallidus, cerebellum, cortex, thalamus, pons and others (from left to right). The rows were sorted according to the row mean expression level (bottom is highest). **C**, **D.** The mean expression level intensity (pink squares) and the entropy (blue diamonds) for each ranked position and for the functional tissues in human (C) and mouse (D).
