# Supplementary material for: Widespread ectopic expression of olfactory receptor genes
Source: BMC Genomics. 2006 May 22;7:121. doi: 10.1186/1471-2164-7-121 (PMC1508154; doi:10.1186/1471-2164-7-121)
Supplement: Additional File 5 — Figures showing ectopic expression of brain specific and spermatogenesis related genes across 48 mouse tissues are shown in Additional file 5 [file 1471-2164-7-121-S5.pdf]

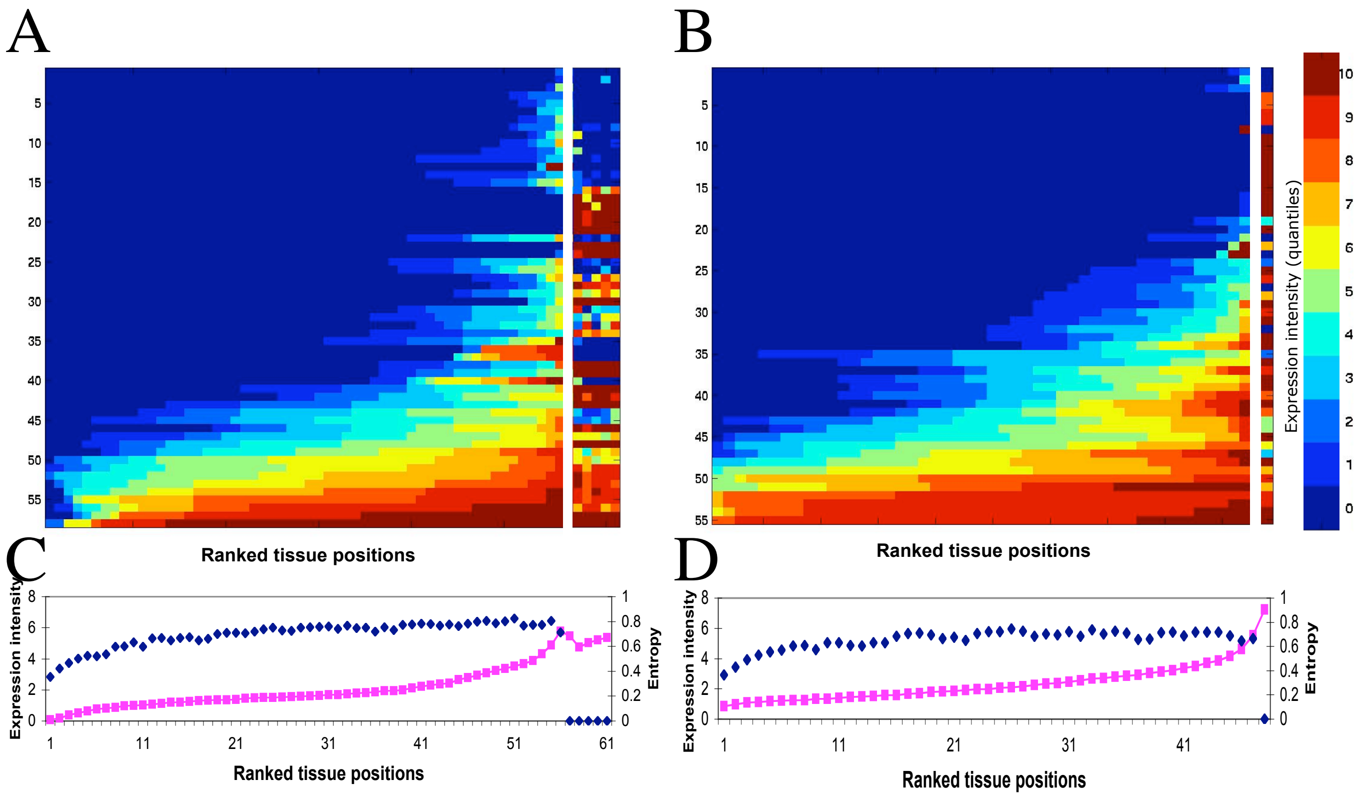

Ranked quantification of spermatogenesis related genes ectopic expression. A, B. Dually sorted matrices show ectopic expression for human (A) and mouse (B) spermatogenesis related genes. For every gene (rows) representing probesets were sorted according to the expression intensity, so the tissue with the highest expression level for that gene is on the right. The separate column(s) on the right represent the functional tissue(s): testis, germinal cells, Leydig cells and testis interstitial tissue from left to right in A and testis in B. The rows were sorted according to the row mean expression level (bottom is highest). **C, D.** The mean expression level intensity (pink squares) and the entropy (blue diamonds) for each ranked position and for the functional tissues in human (C) and mouse (D).
